# *O*-GlcNAcylation of Focal Adhesion Kinase Regulates Cell Adhesion, Migration, and Proliferation via the FAK/AKT Pathway

**DOI:** 10.3390/biom14121577

**Published:** 2024-12-10

**Authors:** Zhiwei Zhang, Tomoya Isaji, Yoshiyuki Oyama, Jianwei Liu, Zhiwei Xu, Yuhan Sun, Tomohiko Fukuda, Haojie Lu, Jianguo Gu

**Affiliations:** 1 Division of Regulatory Glycobiology, Graduate School of Pharmaceutical Sciences, Tohoku Medical and Pharmaceutical University, Sendai 980-0845, Miyagi, Japan; zzw1997117@gmail.com (Z.Z.); joyama0178@gmail.com (Y.O.); 85088681ljw@gmail.com (J.L.); tohokuxu@gmail.com (Z.X.); sunyuhan2@shchildren.com (Y.S.); tfukuda@tohoku-mpu.ac.jp (T.F.); 2 Institute of Molecular Biomembrane and Glycobiology, Tohoku Medical and Pharmaceutical University, 4-4-1 Komatsushima, Aoba-ku, Sendai 981-8558, Miyagi, Japan; 3 Shanghai Cancer Center and Institutes of Biomedical Sciences, Fudan University, Shanghai 200032, China; luhaojie@fudan.edu.cn

**Keywords:** cell adhesion, cellular signaling, focal adhesion kinase, *O*-GlcNAcylation, OGT

## Abstract

Focal Adhesion Kinase (FAK) is a non-receptor tyrosine kinase pivotal in cellular signal transduction, regulating cell adhesion, migration, growth, and survival. However, the regulatory mechanisms of FAK during tumorigenesis and progression still need to be fully understood. Our previous study demonstrated that *O*-GlcNAcylation regulates integrin-mediated cell adhesion. To further elucidate the underlying molecular mechanism, we focused on FAK in this study and purified it from 293T cells. Using liquid chromatography–mass spectrometry (LC-MS/MS), we identified the *O*-GlcNAcylation of FAK at Ser708, Thr739, and Ser886. Compared with wild-type FAK expressed in *FAK*-knockout 293T cells, the FAK mutant, in which Ser708, Thr739, and Ser886 were replaced with Ala, exhibited lower phosphorylation levels of Tyr397 and AKT. Cell proliferation and migration, assessed through MTT and wound healing assays, were significantly suppressed in the FAK mutant cells compared to the wild-type FAK cells. Additionally, the interaction among FAK, paxillin, and talin was enhanced, and cell adhesion was increased in the mutant cells. These data indicate that specific *O*-GlcNAcylation of FAK plays a critical regulatory role in integrin-mediated cell adhesion and migration. This further supports the idea that *O*-GlcNAcylation is essential for tumorigenesis and progression and that targeting the *O*-GlcNAcylation of FAK could offer a promising therapeutic strategy for cancer treatment.

## 1. Introduction

Cell adhesion and migration are fundamental processes in cell biology crucial for development, tissue maintenance, wound healing, and immune responses [1]. These processes are primarily regulated by signaling pathways initiated by cell adhesion receptors, particularly integrins, which are heterodimeric transmembrane glycoproteins. Integrins play a vital role in cancer cell signaling through their interactions with the extracellular matrix (ECM) [2,3,4].

FAK, a non-receptor tyrosine–protein kinase, is central to integrin-mediated signaling, which is pivotal to cellular migration and adhesion dynamics [5]. It interacts with focal adhesion (FA) proteins and is mainly characterized by its autophosphorylation at Tyr397, a critical event that enhances signaling by recruiting Src family kinases and other signaling molecules. Studies in *FAK*-knockout mice have demonstrated that disruptions in both FAK and growth factor signaling pathways, as well as diminished cytoskeletal dynamics at FAs, significantly impair cellular migration [6,7,8,9,10]. Furthermore, inhibiting the formation of the FAK-Src complex increases FA formation, while FAK overexpression enhances cellular migration and invasion, contributing to tumor progression and metastasis [9,11,12,13]. Although not classified as an oncogene, FAK is overexpressed in various tumors, including pancreatic, colorectal, and breast cancer, where it modulates critical processes, such as proliferation and survival, thereby worsening prognosis [14,15,16,17,18,19,20,21,22,23,24,25,26,27]. This underscores FAK’s potential as a therapeutic target, with small molecule inhibitors showing promise in preclinical models and advancing to clinical trials [22,28,29].

Protein glycosylation, a prevalent post-translational modification in eukaryotes, is essential for regulating protein function [30]. Defects in glycosylation can lead to severe conditions, including developmental disorders and muscular dystrophy [31,32]. Among various forms of glycosylation, *O*-GlcNAcylation is particularly notable. This modification, involving the addition of a single N-acetylglucosamine (GlcNAc) molecule to Ser or Thr residues on target intracellular proteins, is facilitated by the enzymes *O*-GlcNAc transferase (OGT) and *O*-GlcNAcase (OGA) [33]. *O*-GlcNAcylation plays a critical role in regulating protein functions, gene expression, the cell cycle, and stress responses [34,35]. It significantly interacts with Ser/Thr phosphorylation, impacting various cellular processes [36,37,38]. This interplay is crucial, as it influences the mechanisms underlying several diseases, including cancer, where elevated levels of *O*-GlcNAcylation promote tumorigenesis through mechanisms involving altered cell adhesion, migration, and immune evasion [39,40,41,42].

Recent studies suggest that *O*-GlcNAcylation promotes malignant phenotypes in certain cancers by stabilizing *Nrf2* and activating the PI3K/AKT pathway [43,44]. Experiments with *OGT* knockdown (KD) in HeLa cell lines have revealed that *O*-GlcNAcylation of essential FA proteins, such as paxillin, talin, and FAK, regulates cell adhesion, migration, and FA complex formation [45]. Interestingly, reduced *O*-GlcNAcylation of these proteins led to significantly increased complex formation with integrin-β1, highlighting *O*-GlcNAcylation’s critical role in regulating integrin-mediated functions and FA complex stability. However, further research is needed to understand how *O*-GlcNAcylation affects FAK and its pathways.

In this study, we purified FAK and identified *O*-GlcNAcylation sites on FAK. LC-MS/MS analysis showed that Ser708, Thr739, and Ser886 of FAK were modified by *O*-GlcNAc. To assess the impact of these modifications on FAK’s biological functions, we introduced an FAK variant lacking these three *O*-GlcNAcylation sites into an *FAK*-knockout 293T cell. The results revealed that *O*-GlcNAcylation is essential for cell proliferation and migration and influences phosphorylation levels of Tyr397 and downstream AKT signaling. Thus, *O*-GlcNAcylation of FAK could be considered a new regulator for integrin-mediated cell signaling, which includes cell adhesion and migration.

## 2. Materials and Methods

### 2.1. Antibodies and Reagents

The following antibodies and reagents were used: FAK Tyr397 (611807) and Paxillin (610052) from BD Transduction Laboratories; *O*-GlcNAc (9875S), Phospho-SAPK/JNK (4688), SAPK/JNK (9252), Phospho-p44/42 MAPK (Erk1/2) (4370), p44/42 MAPK (Erk1/2) (4695), Phospho-AKT (4060), AKT (9272), and peroxidase-conjugated secondary antibody against rabbit (7074S) from Cell Signaling Technology, Danvers, MA, USA; OGT (O6014), Talin (T3287), VSV-G (vesicular stomatitis virus glycoprotein) (V5507), α-tubulin (T6199), and peroxidase-conjugated secondary antibody against mouse IgG (AP124P) from Sigma, St. Louis, MO, USA; peroxidase-conjugated secondary antibody against goat IgG (AB324P) from Merck Millipore, Burlington, MA, USA; Anti-Green Fluorescent Protein (GFP) mAb-Magnetic Beads (MBL, D153-11) and Anti-GFP (Rockland, 600-101-215); Anti-VSV-G-Agarose antibody (A1970) from Sigma-Aldrich; human fibronectin (FN) and doxycycline hyclate (D9891) from Sigma-Aldrich, St. Louis, MO, USA; Coomassie Brilliant Blue (CBB) Stain One Super (Ready to use, 11642-31) from Nacalai Tesque, Kyoto, Japan; and the ABC kit (PK-4000) from Vector Laboratories, Burlingame, CA, USA.

### 2.2. Cell Culture and Cell Lines

The 293T cell line was obtained from RIKEN (Wako, Saitama, Japan) and cultured in DMEM high glucose (Invitrogen, Waltham, MA, USA) containing 10% fetal bovine serum (Gibco, Waltham, MA, USA) at 37 °C with 5% CO_2_. The *FAK*-knockout and *OGT* KD 293T cell lines were established in previous studies in our laboratory [45,46].

### 2.3. Expression Plasmids and Transfection

The expression vector pRKVSV-FAK (mouse FAK) was kindly provided by Dr. Kenneth Yamada (NIDCR/NIH) [47]. GFP was inserted at the N-terminus using in-fusion (Clontech, Mountain View, CA, USA) and cloned into the pENTR D-TOPO vector. Site-directed mutagenesis was performed to replace Ser708, Thr739, and Ser886 with Ala, as identified through MS and confirmed through Sanger sequencing. The mutagenesis primers are listed in Supplementary Table S1. The mutated constructs were transferred into the mammalian expression vector (pcDNA3.1Zeo Rfa) using LR Clonase (Invitrogen) for transient transfection experiments. For plasmid transfection, PEI MAX (Polysciences Inc, Warrington, PA, USA) was used [45]. Before transfection for 16 h, cells were seeded in a 10 cm dish. Plasmid DNA (6 µg) and PEI MAX (1 mg/mL) were each dissolved in 1 mL of Opti-MEM (Gibco), combined after 5 min, mixed, and incubated for 15 min before being added to the cell culture dish. After 6 h of incubation, the medium was replaced with a fresh culture medium, and the cells were incubated for an additional 48 h.

### 2.4. Western Blot and Immunoprecipitation

Cells were washed with pre-cooled PBS and lysed in a lysis buffer (20 mM Tris-HCl, pH 7.4, 150 mM NaCl, 0.1% Triton X-100) containing protease and phosphatase inhibitors (Nacalai Tesque, Kyoto, Japan). The lysate was centrifuged at 12,000× *g* for 10 min at 4 °C, and the supernatant was collected for protein concentration determination using a BCA (Bicinchoninic Acid, Thermo Fisher Scientific, Waltham, MA, USA) protein assay kit (Wako, Osaka, Japan). Proteins were separated using SDS-PAGE and transferred to a PVDF membrane (Millipore, Billerica, MA, USA). The membrane was blocked with 5% non-fat milk in TBS-T for 1 h at room temperature and then incubated with primary antibodies overnight at 4 °C. After washing, the membrane was incubated with horseradish peroxidase (HRP)-conjugated secondary antibodies for 1 h at room temperature. Cell supernatants were incubated with GFP- or VSV-G-conjugated beads at 4 °C for 2 h for immunoprecipitation to isolate GFP- or VSV-G-tagged FAK proteins, followed by Western blot analysis. Original figures can be found in Supplementary Materials.

### 2.5. FAK Purification and MS Analysis of FAK O-GlcNAcylation Sites

For FAK purification, 293T cells were transiently transfected with FAK-VSV-G. After 48 h, approximately 1 × 10^8^ cells were lysed, and FAK was immunoprecipitated using an anti-VSV-G-Agarose antibody. The proteins were separated using SDS-PAGE and stained with CBB, and the target protein band was excised for further processing. The gel was destained with a destaining solution (40% methanol, 10% acetic acid) until the background was transparent for further processing. Protein bands underwent in-gel reduction, alkylation, and destaining, followed by digestion [48].

*O*-GlcNAc peptides were desalted using a C18 tip and analyzed through nanospray LC-MS/MS using an Orbitrap Fusion Tribrid system coupled to an EASY-nano-LC system (Thermo Scientific, Waltham, MA, USA). Peptides were separated on a C18 column (50 cm × 75 μm i.d.) at a flow rate of 200 nL/min. The solvent system included 0.1% formic acid in water (Solvent A) and 90% acetonitrile with 0.1% formic acid (Solvent B). The gradient ran over 1 h: 1–20% B over 38 min, increasing to 30% B over 7 min, then to 90% B over 3 min, held for 7 min, reduced to 1% in 10 s, and maintained at 1% for the final 4.83 min. Data were analyzed using Byonic software 4.3. Search parameters included a mass tolerance of ±10 ppm for precursors and ±20 ppm for product ions, with HCD fragmentation. Specificity for trypsin with ≤3 missed cleavages was set. The database search was conducted against a mouse FAK1 protein database from UniProt, European Bioinformatics Institute (EBI), Hinxton, Cambridge, UK. Variable modifications included methionine oxidation and phosphorylation of Ser/Thr/Tyr, with carbamidomethylation of cysteine as a fixed modification. *O*-GlcNAc was also included as a variable modification. Identifications were considered successful with a Byonic score above 150 and logProb (logarithmic probability) above 1.

### 2.6. Cell Adhesion Assay

First, 96-well Cell Carrier plates (PerkinElmer Life Sciences, Waltham, MA, USA) were coated with FN (10 µg/mL) overnight at 4 °C. Transiently transfected cells were reseeded into the plates at a density of 2 × 10^4^ cells/well and incubated at 37 °C for 30 min. Non-adherent cells were gently removed with PBS. Cells were fixed with 4% formaldehyde (Merck Millipore) for 10 min at room temperature and stained with DAPI (4′,6-diamidino-2-phenylindole, Dojindo) for 5 min. Images were captured using the Operetta CLS (PerkinElmer Life Sciences), and the number of nuclei per well was calculated using Harmony software 5.1 (PerkinElmer Life Sciences, Waltham, MA, USA).

### 2.7. Wound Healing Assay

Cells were seeded in six-well plates and grown to confluence. Wounds were created using a 200 µL pipette tip. Cells were washed with PBS and incubated in DMEM with 1% FBS. Images were captured immediately after wounding and at regular intervals during incubation using a phase-contrast microscope (Olympus, Tokyo, Japan). The relative migration distance of each group was calculated using ImageJ software 1.51o.

### 2.8. Cell Proliferation

Cells were seeded in 96-well plates at 10^3^ cells/well density and cultured for different time points (24, 48, and 72 h). Then, 10 µL of 5 mg/mL MTT (3-[4,5-dimethylthiazol-2-yl]-2,5-diphenyltetrazolium bromide; Dojindo, Kumamoto, Japan) was added to each well, and the cells were incubated for another 4 h at 37 °C. The medium was discarded, and 100 µL of DMSO (Wako) was added to each well. Absorbance at 490 nm was measured using a microplate reader (SpectraMax^®^ iD5; Molecular Devices, San Jose, CA, USA).

### 2.9. Statistical Analysis

All experimental data are presented as mean ± SD. Statistical analysis was performed using a two-tailed unpaired Student’s *t*-test or analysis of variance (ANOVA) with GraphPad Prism 5.0 software (GraphPad Software Inc., La Jolla, CA, USA). Significance levels were defined as follows: * *p* < 0.05; ** *p* < 0.01; *** *p* < 0.001. No significance levels are indicated as ns (no significance), *p* > 0.05.

## 3. Results

### 3.1. OGT KD Reduces FAK O-GlcNAcylation in 293T Cells

**Figure 1:**
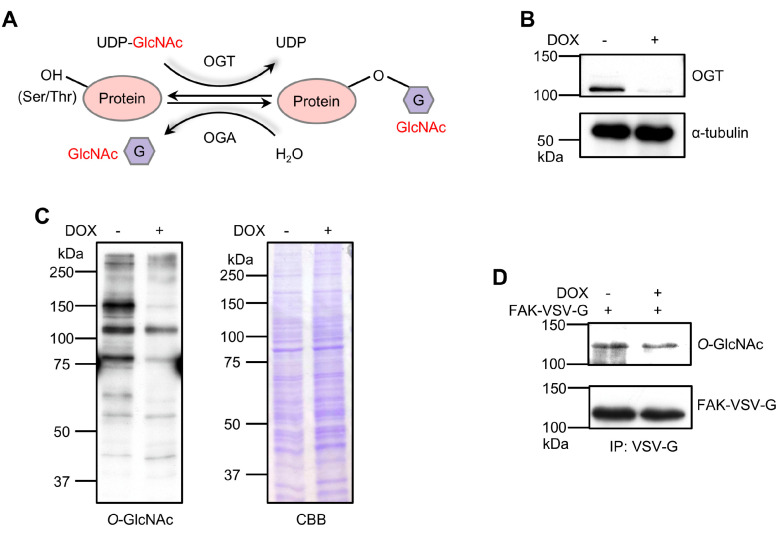
Established *O*-GlcNAcylation KD 293T cell model and confirmed FAK modification by *O*-GlcNAc. (**A**) A schematic diagram illustrating the catalytic reaction of *O*-GlcNAcylation. (**B**) *OGT* KD 293T cells described in the Materials and Methods were treated with 5 μg/mL of DOX for 72 h, with an untreated group as the control. OGT levels were detected through immunoblot. Tubulin was a loading control. Western blot original images can be found in Figure S1. (**C**) The *O*-GlcNAcylation levels in cell lysates of DOX-dependent *OGT* KD 293T cells were validated by the anti-*O*-GlcNAcylation antibody. CBB staining was performed as a loading control. (**D**) VSV-G-tagged FAK was transfected into both control and KD cells. Further immunoprecipitation of cell lysates was performed using a VSV-G antibody, followed by immunoblotting to detect the *O*-GlcNAcylation levels of VSV-G-tagged FAK.

*O*-GlcNAcylation plays a crucial role in regulating various cellular processes [35]. This reversible reaction, catalyzed by OGT and OGA, involves adding and removing GlcNAc moieties from the hydroxyl groups of Ser/Thr residues (Figure 1A). Previously, we demonstrated that *O*-GlcNAcylation of FA proteins regulates cell migration, adhesion, and FA dynamics in HeLa cells [45]. However, the specific proteins influenced by *O*-GlcNAcylation and the critical sites of *O*-GlcNAcylation that are important for these phenotypes remain unclear. To further explore the mechanisms of *O*-GlcNAcylation in FAs, we utilized 293T cells due to their high capacity for exogenous protein expression. These cells have been widely used in significant studies on FAK and integrin signaling, as well as their effects on cell motility and proliferation [49,50,51,52]. Recognizing FAK as a critical molecule in regulating FAs, we specifically focused on the *O*-GlcNAcylation of FAK. As shown in Figure 1B, the expression of endogenous OGT was almost eliminated in *OGT* KD cells. Additionally, significant suppression of *O*-GlcNAcylation was observed in DOX-induced *OGT* KD 293T cells (Figure 1C), consistent with previous observations in HeLa cells [45]. The KD did not affect protein levels, as confirmed by CBB staining. Subsequently, FAK was transiently transfected into both cell groups. As expected, the *O*-GlcNAcylation level of FAK in *OGT* KD cells was significantly reduced (Figure 1D). These results suggest that the DOX-induced *OGT* KD system in 293T cells is suitable for examining FAK *O*-GlcNAcylation functions.

### 3.2. Identification of Specific O-GlcNAcylation Sites on FAK and Construction of Mutant

**Figure 2:**
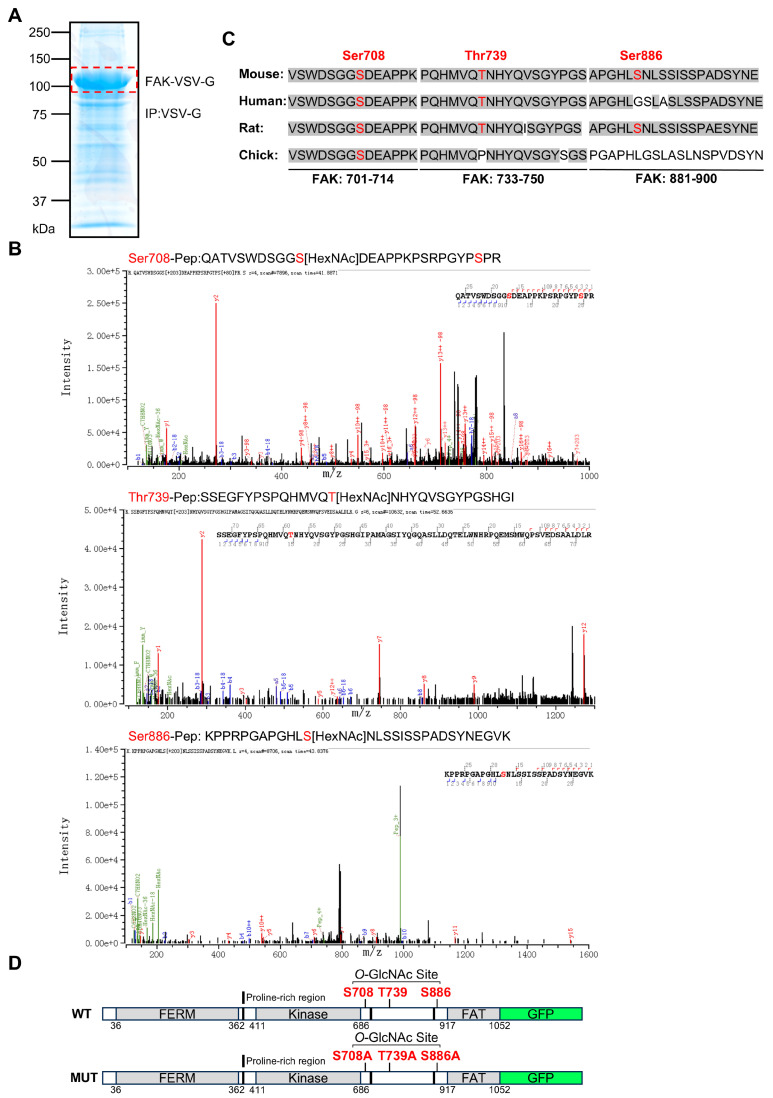
Identification of *O*-GlcNAcylation sites on FAK through LC-MS/MS. (**A**) VSV-G-tagged mouse FAK was transfected into 293T cells, and cell lysates were subjected to immunoprecipitation and separated using 7.5% SDS-PAGE gel. The FAK-VSV-G band (red dashed line) was cut off for LC-MS analysis. (**B**) The sites for determining FAK *O*-GlcNAcylation were mapped using MS to detect *O*-GlcNAcylation sites on FAK. *O*-GlcNAcylation peptides were analyzed through LC-MS/MS. Analysis with the Byonic software identified *O*-GlcNAcylation on three peptides, as indicated. The Ser708, Thr739, and Ser886 sites were marked in red. The phosphorylation site at Ser722 was also detected and shown in red. (**C**) Sequence alignments of FAK at Ser708, Thr739, and Ser886, along with adjacent sequences from different species with conserved serine/threonine residues, were highlighted in red. (**D**) Schematic representation of the wild-type and mutant plasmids utilized in this study. From the N-terminus to the C-terminus are the FERM domain, the kinase domain, the FAT domain, and the GFP tag, where the three black bars represent three proline-rich regions (PRR1, PRR2, PRR3). The MUT indicates that the Ser or Thr at Ser708, Thr739, and Ser886 sites were replaced with Ala through site-directed mutagenesis.

Specific *O*-GlcNAcylation sites on FAK were predicted using a database [53]. The prediction algorithm identified two putative *O*-GlcNAcylation sites on the kinase domain, while the C-terminal proline-rich region and the focal adhesion targeting (FAT) domain contained twenty-eight and two putative *O*-GlcNAcylation sites, respectively. This suggests that the region flanked by the kinase domain and the FAT domain, containing two proline sequences, has numerous predicted modification sites (details are provided in the Supplementary Materials). To determine the presence of *O*-GlcNAcylation on these predicted tryptic peptides, we attempted to detect their precise molecular weights using LC-MS/MS analysis. We transfected 293T cells with the FAK expression vector and purified the recombinant protein via immunoprecipitation using a VSV-G-tag (Figure 2A). Following in-gel digestion and single-step MS identification of the complete glycopeptides, LC-MS analysis identified Ser708, Thr739, and Ser886 as the *O*-GlcNAcylation sites within the C-terminal proline-rich region (Figure 2B). We also identified the phosphorylation site of FAK at Ser722. Sequence comparisons across different species revealed that these three residues are relatively conserved in FAK (Figure 2C). To confirm the functional significance of these sites, we performed site-directed mutagenesis, substituting the potential *O*-GlcNAcylation sites (Ser708, Thr739, and Ser886) with alanine to create the mutant. A GFP tag was also inserted to assess cell transfection efficiency (Figure 2D).

### 3.3. Confirming the Importance of O-GlcNAcylation Sites in FAK

**Figure 3:**
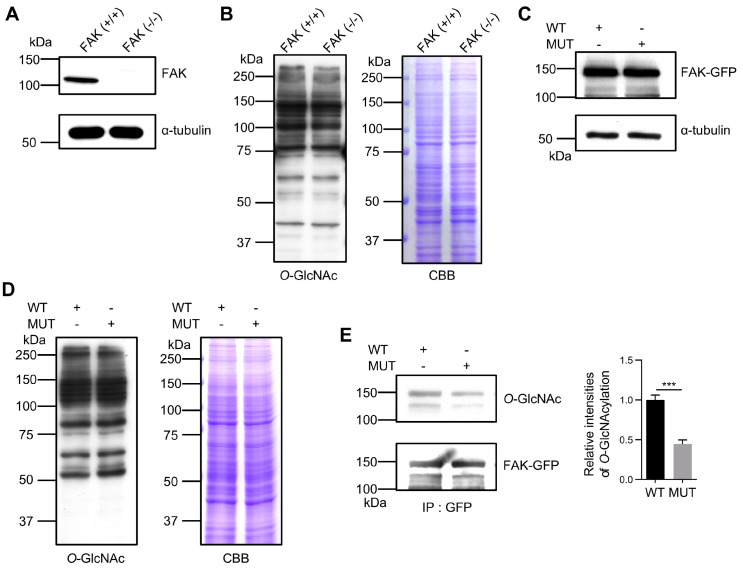
Influences of *O*-GlcNAcylation on FAK in the mutant. Cell lysates were extracted from 293T and 293T FAK-KO cells and analyzed through Western blotting with anti-FAK or anti-*O*-GlcNAc antibodies to detect FAK (**A**) and *O*-GlcNAc modification levels (**B**). α-Tubulin or CBB staining was used as the loading control. (**C**,**D**) Wild-type (WT) and mutant (MUT) FAK plasmids were transfected into the 293T FAK-KO cells, and the expression levels of FAK and *O*-GlcNAcylation were Western blotted with anti-FAK (**C**) or anti-*O*-GlcNAc antibodies (**D**). α-Tubulin or CBB staining as the loading control. (**E**) Cell lysates were immunoprecipitated using anti-GFP magnetic beads, followed by Western blotting with anti-*O*-GlcNAc and anti-GFP antibodies. The relative ratio of *O*-GlcNAcylated FAK to total FAK was normalized to 1.0. Experiments were independently repeated at least three times. Values represent mean ± SD. *** *p* < 0.001.

To further validate the modification of these three sites through *O*-GlcNAcylation and to explore their functions, we employed the 293T FAK-KO cell line to eliminate interference from endogenous FAK (Figure 3A). The FAK-KO did not significantly affect the *O*-GlcNAcylation levels in the cell lysate (Figure 3B). We reintroduced wild-type (WT) and mutant (MUT) FAK expression plasmids into the FAK-KO 293T cell line. Immunoblotting demonstrated that both constructs were expressed at similar levels (Figure 3C) and did not affect the total *O*-GlcNAcylation levels in the cell lysates (Figure 3D). The immunoprecipitant of the GFP antibody followed by immunoblotting revealed a significant reduction in *O*-GlcNAcylation on the MUT compared to the WT (Figure 3E). These results conclusively demonstrate that Ser708, Thr739, and Ser886 are the primary *O*-GlcNAcylation sites on FAK, which might underscore their critical roles in FAK biological functions. Of course, we cannot exclude the possibility of having other *O*-GlcNAcylation sites on FAK, which remains for further study.

### 3.4. FAK O-GlcNAcylation Modulates Cell Adhesion, Migration, and Proliferation

**Figure 4:**
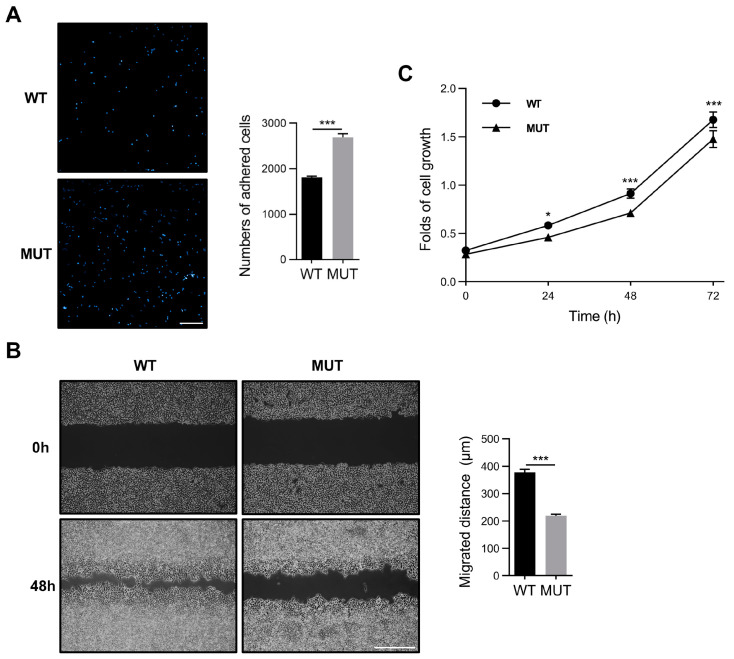
Effects of FAK *O*-GlcNAcylation on cell adhesion, migration and proliferation. (**A**) Equal numbers of WT and MUT cells were seeded into FN-coated 96-well plates for 30 min. Non-adherent cells were washed using PBS. The adhered cells were then fixed with 4% paraformaldehyde and stained with DAPI for nuclear visualization. Representative fields were captured using a fluorescence microscope, and cells were counted. Scale bar, 200 μm. Values represent the mean ± SD (*n* = 5). *** *p* < 0.001. (**B**) Transfected cells were seeded into six-well plates. When the cells reached over 90% confluence, a 200 μL pipette tip was used to scratch each well to create a wound. Images were taken at 0 and 48 h using a phase-contrast microscope. Migration distances were evaluated using ImageJ. Experiments were independently repeated three times. Scale bar, 200 μm. Values represent the mean ± SD (*n* = 3). *** *p* < 0.001. (**C**) Transfected WT and MUT cells were cultured in DMEM with 5% FBS and seeded into 96-well plates. At designated time points (24, 48, 72 h), cell numbers were measured using the MTT assay. Experiments were independently repeated three times. Values represent the mean ± SD (*n* = 05); * *p* < 0.05; *** *p* < 0.001.

*O*-GlcNAcylation and FAK play critical roles in tumor cell growth and metastasis [14,54]. Additionally, the *O*-GlcNAcylation of FA proteins, including FAK, is crucial for cellular processes, such as cell migration and proliferation [45]. Therefore, we investigated the impact of the *O*-GlcNAcylation of FAK on cellular functions, including cell adhesion, migration, and proliferation. First, WT and MUT FAK plasmids were transfected into FAK-KO 293T cells for subsequent experiments. At approximately 48 h post-transfection, equal amounts of cells (2 × 10^4^) were seeded into FN-coated 96-well plates for a 30 min cell adhesion assay. The MUT cells exhibited more adhesion than the WT cells (Figure 4A), which is consistent with our previous observation that decreased total cellular *O*-GlcNAcylation levels enhance cell adhesion [45]. Conversely, the wound healing assay showed that MUT cells had a lower migratory capacity than WT cells (Figure 4B). In addition, we conducted a cell proliferation assay. We found that MUT cells showed lower proliferation capacities than WT cells, suggesting that the reduced cell migration observed in the wound healing assay predominantly reflects decreased migratory capacity but may also be marginally influenced by cell proliferation (Figure 4C). These findings suggest that *O*-GlcNAcylation of FAK significantly influences cellular functions, including cell adhesion, migration, and proliferation.

### 3.5. FAK O-GlcNAcylation Affects Tyr397 Phosphorylation and FA Complex Formation

**Figure 5:**
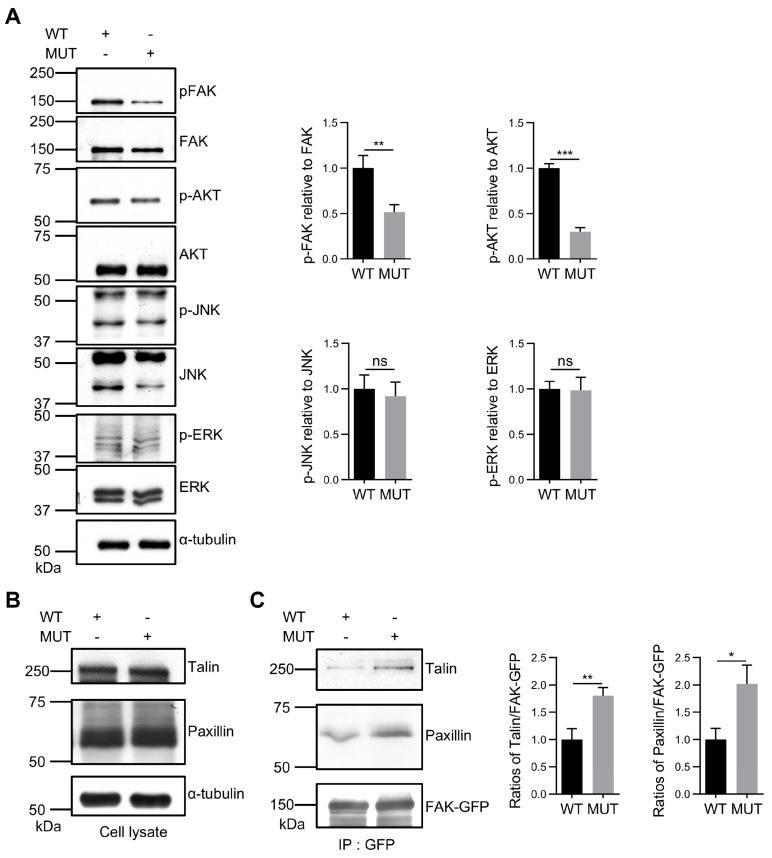
Effects of FAK *O*-GlcNAcylation on cellular signaling and complex formation in FAs. (**A**) Cell lysates were subjected to Western blot analysis using the indicated antibodies to detect phosphorylated Tyr397-FAK, total FAK, p-ERK, total ERK, p-JNK, total JNK, and phosphorylated Akt and total Akt expression levels. The relative expression level of each phosphorylated form vs. the total form was calculated using ImageJ software 1.51o based on the intensity of the phosphorylated form relative to the total form. Values represent mean ± SD. ** *p* < 0.01; *** *p* < 0.001. (**B**) Western blot analysis used the indicated antibodies to detect paxillin and talin expression levels in cell lysates. α-tubulin was used as the loading control. (**C**) Cell lysates were immunoprecipitated using anti-GFP magnetic beads. The immunoprecipitates were Western blotted with the indicated antibodies to detect paxillin and talin levels. Experiments were independently repeated at least three times. The ratio of paxillin or talin intensity vs. the total GFP-FAK in 293T FAK-KO cells expressing WT FAK was 1.0. Experiments were independently repeated at least three times. Values represent mean ± SD. * *p* < 0.05; ** *p* < 0.01. ns, no significance.

We focused on cellular signaling to elucidate the underlying mechanisms for the effects of FAK *O*-GlcNAcylation on cellular functions. It is well-known that Tyr397 is a pivotal phosphorylation site, and it is recognized as the primary and initial site for FAK-mediated signaling cascades [55,56]. Immunoblot analysis revealed that the ablation of *O*-GlcNAcylation on FAK decreased Tyr397 phosphorylation levels and reduced downstream AKT activation (Figure 5A), which may subsequently downregulate cellular migration and proliferation, as observed in Figure 4. In contrast, the activation of downstream ERK and JNK pathways remained unaffected.

The formation of FAs by FAK with paxillin and talin is essential for the dynamic multiprotein complexes that regulate cell behaviors, such as cell adhesion and cell migration [57,58,59]. To assess the formation of FA complexes on FN-coated substrates, we conducted immunoblotting with anti-paxillin and anti-talin antibodies on FAK immunocomplexes. The total expression levels of paxillin and talin did not differ significantly between the WT and MUT cell lysates (Figure 5B), but their levels in FAK immunoprecipitant were increased in the MUT cells compared to the WT cells (Figure 5C). These findings underscore the roles of *O*-GlcNAcylation at Ser708, Thr739, and Ser886 on FAK in the formation of FA complexes, which in turn regulates the activation of the FAK/AKT signaling cascade and influences cell migration and proliferation.

## 4. Discussion

**Figure 6:**
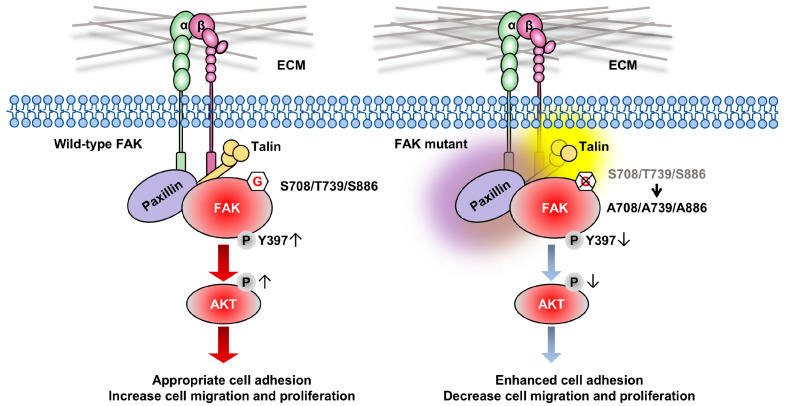
Schematic working model for regulating FAK-mediated cellular signaling and cell behaviors by *O*-GlcNAcylation. In normal conditions, integrin-mediated cell adhesion upregulates Tyr397 phosphorylation of FAK and downstream signaling, which induces appropriate cell adhesion to promote cell migration and proliferation. Ablation of *O*-GlcNAcylation on the three sites of FAK decreases its Tyr397 phosphorylation. It enhances FA formation through the upregulation of FAK, talin, and paxillin complex formation, which in turn suppresses cell migration and proliferation.

This study elucidated that FAK undergoes *O*-GlcNAcylation at specific sites (Ser708, Thr739, and Ser886) and that *O*-GlcNAcylation of FAK has essential biological functions. The ablation of *O*-GlcNAcylation at these three sites on FAK enhanced cell adhesion by promoting stronger interactions among FAK, paxillin, and talin, which conversely suppressed cell migration, supporting the notion that appropriate cell adhesion is better for cell migration [60]. In addition, FAK *O*-GlcNAcylation contributes to Tyr397 phosphorylation of FAK, a most critical and central phosphorylation site for integrin-mediated signaling and its downstream AKT activation. These observations suggest that the *O*-GlcNAcylation of FAK is similar to its phosphorylation of FAK in that it dynamically participates in modulating cell adhesion, migration, and proliferation (Figure 6).

Cell migration is essential for organismal development and is closely linked to various pathological conditions, including cancer and immune dysfunctions [61]. Integrins connect cells to the ECM through FA, a critical component [62]. Studies on FAK-deficient cells have revealed that the absence of FAK leads to increased FAs and decreased cell motility, highlighting FAK’s crucial role in FA turnover during cell migration [10]. A pivotal event in this dynamic is the autophosphorylation of FAK at Tyr397, which facilitates further phosphorylation of proteins, such as Src and paxillin, and regulates the entire assembly of FAs [63,64,65,66]. The FAT domain is essential for its localization at FAs and for promoting the tyrosine phosphorylation of downstream substrates like paxillin, which is crucial for FA turnover and efficient cell migration [67]. Additionally, the phosphorylation of paxillin plays a vital role in FAK-dependent FA dynamics [68]. Recent research, along with historical findings, have underscored the significance of interactions within FAK’s C-terminal proline-rich domain in modulating its interactions with substrates, such as p130^Cas^ and cortactin [69,70,71,72]. However, excessive binding between p130^Cas^ and cortactin in the proline-rich domain might hinder essential dissociations between talin and FAK, as well as paxillin and FAK, thus impeding FA turnover [69]. These inhibitions significantly affect the regulation of FA dynamics, emphasizing the necessity of balanced interactions to maintain cellular motility and integrity.

Because phosphorylation and *O*-GlcNAcylation modify the same Ser and Thr residues, *O*-GlcNAcylation is presumably believed to influence the phosphorylation of FAK, which is known to mitigate excessive protein interactions [73]. The present study suggests that the *O*-GlcNAcylation of FAK could downregulate the dynamics of FA turnover by affecting the complex formation, although the competition between phosphorylation and *O*-GlcNAcylation was not directly observed in this study. Curiously, a previous MS analysis of purified chicken FAK identified phosphorylation at Ser708 [74], a site that was found to be occupied by *O*-GlcNAcylation in mouse FAK in this study. Taken together, these studies support the notion that phosphorylation and *O*-GlcNAcylation exist in competition for modification. Additionally, the proline-rich C-terminal domain contains numerous phosphorylation sites for Ser and Thr residues, further underscoring the multifaceted regulation of this kinase [75]. Specifically, the phosphorylation of Ser732 by Rho-associated kinase, which is essential for cell migration stimulated by vascular endothelial growth factor, illustrates the dynamic role of these modifications in cellular motility [76]. The phosphorylation of Ser722 by glycogen synthase kinase 3 and protein phosphatase 1 is critical in coordinating cell adhesion and migration. This phosphorylation event at Ser722 attenuates FAK’s signaling activity, which in turn facilitates cell diffusion and migration [77]. Coincidentally, the phosphorylation of Ser722 was also identified in our mass spectrometry analysis (Figure 2B), further validating our data. These observations align with our findings that the *O*-GlcNAcylation of the C-terminal of FAK plays an essential role in regulating FA turnover, cell motility, and proliferation.

It is well-known that both *O*-GlcNAcylation and FAK are involved in several pathologies, including diabetes, neurodegenerative diseases, and, particularly, cancer [78]. Our study clearly shows that the modification of FAK by *O*-GlcNAcylation potentially regulates FAK-mediated cellular signaling and dynamics of FAs, which are crucial for cell adhesion and migration during cancer progression and development. OGT is vital for cell division, as its inhibition results in cell death, thereby rendering it unsuitable as a target for diseases, including cancer therapy [79]. Therefore, detecting specific *O*-GlcNAcylation sites on FAK could be an alternative approach for targeting diseases. In addition, FAK plays a crucial role in regulating the sialylation of *N*-glycans on the cell surface, which is essential for cancer migration and metastasis [46]. The interplay between intracellular *O*-GlcNAcylation and surface sialylation may open new avenues for understanding cancer mechanisms.

## 5. Conclusions

This study discovered that FAK is *O*-GlcNAcylated at specific sites—Ser708, Thr739, and Ser886. This post-translational modification is crucial for regulating cell adhesion, migration, and proliferation. *O*-GlcNAcylation of FAK influences FA turnover and enhances phosphorylation at Tyr397, a key player in integrin-mediated signaling. This, in turn, activates downstream pathways like AKT, affecting cellular movement and the dynamics of the cytoskeleton. Our findings highlight the complex interaction between *O*-GlcNAcylation and phosphorylation in fine-tuning FAK function.

Beyond its established role in FA signaling, FAK *O*-GlcNAcylation emerges as an essential link between intracellular glycosylation and pathological processes, such as cancer progression and metastasis. Identifying these specific *O*-GlcNAcylation sites provides new opportunities for therapeutic interventions in diseases with abnormal FAK activity and glycosylation. Moreover, the interplay between intracellular *O*-GlcNAcylation and extracellular *N*-glycan sialylation reveals a complex regulatory network that significantly impacts cancer cell adhesion, invasion, and metastasis. This offers promising avenues for developing targeted therapeutic strategies.

## Data Availability

The data used to support this study’s findings are available from the corresponding author upon reasonable request.

## Supplementary Materials

The following supporting information can be downloaded at https://www.mdpi.com/article/10.3390/biom14121577/s1. Figure S1: original Western blot. Table S1: Sequences of Primers Designed for Mutagenesis.
