# Supplementary material for: O-GlcNAcylation of Focal Adhesion Kinase Regulates Cell Adhesion, Migration, and Proliferation via the FAK/AKT Pathway
Source: Biomolecules. 2024 Dec 10;14(12):1577. doi: 10.3390/biom14121577 (PMC11674061; doi:10.3390/biom14121577)
Supplement: Supplementary file 1 [file biomolecules-14-01577-s001.zip › Supplementary Materials.docx]

**Supplementary Information**

**1. Supplementary Table S1**

Sequences of Primers Designed for Mutagenesis

| Primer Name | Sequence (5’-3’) |
| --- | --- |
| mFAK-S708A-F | CTGGAGGGGCAGATGAAGCACCAACCCAAGCCC |
| mFAK-S708A-R | CTTCATCTGCCCCTCCAGAGTCCCAGGACAC |
| mFAK-T739A-F | GTCCAGGCAAATCACTACCAGGTCTCTGGCTACCC |
| mFAK-T739A-R | GTAGTGATTTGCCTGGACCATGTGCTGTGGGC |
| mFAK-S886A-F | CCACCTAGCAAACCTGTCCAGCATCAGCAGCC |
| mFAK-S886A-R | GACAGGTTTGCTAGGTGGCCAGGTGCTCCAG |

**2. Predictions of O-GlcNAcylation sites in FAK**

Only sites with scores exceeding 0.5 are predicted to be Glycosylated and are annotated as '#POSITIVE' in the comment field. The sites obtained by LC/MS analysis are marked in red font.

##source-version NetOGlyc 4.0.0.13

##FAK

#seqname source feature start end score strand frame comment

SEQUENCE netOGlyc-4.0.0.13 CARBOHYD 13 13 0.859118 . . #POSITIVE

SEQUENCE netOGlyc-4.0.0.13 CARBOHYD 15 15 0.951956 . . #POSITIVE

SEQUENCE netOGlyc-4.0.0.13 CARBOHYD 16 16 0.934563 . . #POSITIVE

SEQUENCE netOGlyc-4.0.0.13 CARBOHYD 17 17 0.974143 . . #POSITIVE

SEQUENCE netOGlyc-4.0.0.13 CARBOHYD 18 18 0.83771 . . #POSITIVE

SEQUENCE netOGlyc-4.0.0.13 CARBOHYD 20 20 0.646361 . . #POSITIVE

SEQUENCE netOGlyc-4.0.0.13 CARBOHYD 24 24 0.591539 . . #POSITIVE

SEQUENCE netOGlyc-4.0.0.13 CARBOHYD 29 29 0.5 . . #POSITIVE

**FERM Domain**

SEQUENCE netOGlyc-4.0.0.13 CARBOHYD 45 45 0.0494282 . .

SEQUENCE netOGlyc-4.0.0.13 CARBOHYD 46 46 0.0581469 . .

SEQUENCE netOGlyc-4.0.0.13 CARBOHYD 47 47 0.110512 . .

SEQUENCE netOGlyc-4.0.0.13 CARBOHYD 50 50 0.0312081 . .

SEQUENCE netOGlyc-4.0.0.13 CARBOHYD 51 51 0.0704144 . .

SEQUENCE netOGlyc-4.0.0.13 CARBOHYD 54 54 0.0679914 . .

SEQUENCE netOGlyc-4.0.0.13 CARBOHYD 62 62 0.0851732 . .

SEQUENCE netOGlyc-4.0.0.13 CARBOHYD 74 74 0.0593664 . .

SEQUENCE netOGlyc-4.0.0.13 CARBOHYD 88 88 0.0289711 . .

SEQUENCE netOGlyc-4.0.0.13 CARBOHYD 92 92 0.0984129 . .

SEQUENCE netOGlyc-4.0.0.13 CARBOHYD 105 105 0.311522 . .

SEQUENCE netOGlyc-4.0.0.13 CARBOHYD 106 106 0.19367 . .

SEQUENCE netOGlyc-4.0.0.13 CARBOHYD 138 138 0.0219074 . .

SEQUENCE netOGlyc-4.0.0.13 CARBOHYD 143 143 0.0713292 . .

SEQUENCE netOGlyc-4.0.0.13 CARBOHYD 153 153 0.0517896 . .

SEQUENCE netOGlyc-4.0.0.13 CARBOHYD 179 179 0.0665352 . .

SEQUENCE netOGlyc-4.0.0.13 CARBOHYD 192 192 0.16807 . .

SEQUENCE netOGlyc-4.0.0.13 CARBOHYD 210 210 0.396795 . .

SEQUENCE netOGlyc-4.0.0.13 CARBOHYD 214 214 0.216275 . .

SEQUENCE netOGlyc-4.0.0.13 CARBOHYD 219 219 0.32384 . .

SEQUENCE netOGlyc-4.0.0.13 CARBOHYD 227 227 0.248931 . .

SEQUENCE netOGlyc-4.0.0.13 CARBOHYD 239 239 0.0199329 . .

SEQUENCE netOGlyc-4.0.0.13 CARBOHYD 248 248 0.0348019 . .

SEQUENCE netOGlyc-4.0.0.13 CARBOHYD 264 264 0.00533628 . .

SEQUENCE netOGlyc-4.0.0.13 CARBOHYD 265 265 0.0114547 . .

SEQUENCE netOGlyc-4.0.0.13 CARBOHYD 269 269 0.0188729 . .

SEQUENCE netOGlyc-4.0.0.13 CARBOHYD 281 281 0.0850894 . .

SEQUENCE netOGlyc-4.0.0.13 CARBOHYD 284 284 0.0652537 . .

SEQUENCE netOGlyc-4.0.0.13 CARBOHYD 291 291 0.160452 . .

SEQUENCE netOGlyc-4.0.0.13 CARBOHYD 301 301 0.0887246 . .

SEQUENCE netOGlyc-4.0.0.13 CARBOHYD 305 305 0.18167 . .

SEQUENCE netOGlyc-4.0.0.13 CARBOHYD 307 307 0.101795 . .

SEQUENCE netOGlyc-4.0.0.13 CARBOHYD 328 328 0.082111 . .

SEQUENCE netOGlyc-4.0.0.13 CARBOHYD 330 330 0.136789 . .

SEQUENCE netOGlyc-4.0.0.13 CARBOHYD 333 333 0.048304 . .

SEQUENCE netOGlyc-4.0.0.13 CARBOHYD 335 335 0.02116 . .

SEQUENCE netOGlyc-4.0.0.13 CARBOHYD 355 355 0.153634 . .

SEQUENCE netOGlyc-4.0.0.13 CARBOHYD 357 357 0.0839275 . .

**FERM Domain ↔ Kinase Domain**

SEQUENCE netOGlyc-4.0.0.13 CARBOHYD 372 372 0.470589 . .

SEQUENCE netOGlyc-4.0.0.13 CARBOHYD 379 379 0.755566 . . #POSITIVE

SEQUENCE netOGlyc-4.0.0.13 CARBOHYD 386 386 0.682403 . . #POSITIVE

SEQUENCE netOGlyc-4.0.0.13 CARBOHYD 390 390 0.84801 . . #POSITIVE

SEQUENCE netOGlyc-4.0.0.13 CARBOHYD 392 392 0.601429 . . #POSITIVE

SEQUENCE netOGlyc-4.0.0.13 CARBOHYD 394 394 0.481673 . .

SEQUENCE netOGlyc-4.0.0.13 CARBOHYD 406 406 0.570016 . . #POSITIVE

SEQUENCE netOGlyc-4.0.0.13 CARBOHYD 408 408 0.5 . . #POSITIVE

**Kinase Domain**

SEQUENCE netOGlyc-4.0.0.13 CARBOHYD 411 411 0.809173 . . #POSITIVE

SEQUENCE netOGlyc-4.0.0.13 CARBOHYD 412 412 0.542753 . . #POSITIVE

SEQUENCE netOGlyc-4.0.0.13 CARBOHYD 443 443 0.0247449 . .

SEQUENCE netOGlyc-4.0.0.13 CARBOHYD 455 455 0.0215102 . .

SEQUENCE netOGlyc-4.0.0.13 CARBOHYD 460 460 0.0406896 . .

SEQUENCE netOGlyc-4.0.0.13 CARBOHYD 461 461 0.128478 . .

SEQUENCE netOGlyc-4.0.0.13 CARBOHYD 463 463 0.0303016 . .

SEQUENCE netOGlyc-4.0.0.13 CARBOHYD 474 474 0.0603773 . .

SEQUENCE netOGlyc-4.0.0.13 CARBOHYD 491 491 0.0113832 . .

SEQUENCE netOGlyc-4.0.0.13 CARBOHYD 503 503 0.014298 . .

SEQUENCE netOGlyc-4.0.0.13 CARBOHYD 509 509 0.112734 . .

SEQUENCE netOGlyc-4.0.0.13 CARBOHYD 517 517 0.0876697 . .

SEQUENCE netOGlyc-4.0.0.13 CARBOHYD 522 522 0.0384412 . .

SEQUENCE netOGlyc-4.0.0.13 CARBOHYD 531 531 0.0650927 . .

SEQUENCE netOGlyc-4.0.0.13 CARBOHYD 532 532 0.0457578 . .

SEQUENCE netOGlyc-4.0.0.13 CARBOHYD 539 539 0.0187932 . .

SEQUENCE netOGlyc-4.0.0.13 CARBOHYD 555 555 0.0600556 . .

SEQUENCE netOGlyc-4.0.0.13 CARBOHYD 556 556 0.0452057 . .

SEQUENCE netOGlyc-4.0.0.13 CARBOHYD 568 568 0.160836 . .

SEQUENCE netOGlyc-4.0.0.13 CARBOHYD 574 574 0.0595894 . .

SEQUENCE netOGlyc-4.0.0.13 CARBOHYD 575 575 0.0448707 . .

SEQUENCE netOGlyc-4.0.0.13 CARBOHYD 580 580 0.0660013 . .

SEQUENCE netOGlyc-4.0.0.13 CARBOHYD 593 593 0.111363 . .

SEQUENCE netOGlyc-4.0.0.13 CARBOHYD 600 600 0.0352308 . .

SEQUENCE netOGlyc-4.0.0.13 CARBOHYD 601 601 0.095037 . .

SEQUENCE netOGlyc-4.0.0.13 CARBOHYD 603 603 0.0265736 . .

SEQUENCE netOGlyc-4.0.0.13 CARBOHYD 650 650 0.255775 . .

SEQUENCE netOGlyc-4.0.0.13 CARBOHYD 653 653 0.0805852 . .

SEQUENCE netOGlyc-4.0.0.13 CARBOHYD 656 656 0.176454 . .

SEQUENCE netOGlyc-4.0.0.13 CARBOHYD 664 664 0.232889 . .

**Kinase Domain ↔ FAT** **Domain**

SEQUENCE netOGlyc-4.0.0.13 CARBOHYD 670 670 0.367418 . .

SEQUENCE netOGlyc-4.0.0.13 CARBOHYD 677 677 0.285747 . .

SEQUENCE netOGlyc-4.0.0.13 CARBOHYD 678 678 0.198237 . .

SEQUENCE netOGlyc-4.0.0.13 CARBOHYD 695 695 0.876802 . . #POSITIVE

SEQUENCE netOGlyc-4.0.0.13 CARBOHYD 700 700 0.864845 . . #POSITIVE

SEQUENCE netOGlyc-4.0.0.13 CARBOHYD 702 702 0.889594 . . #POSITIVE

SEQUENCE netOGlyc-4.0.0.13 CARBOHYD 705 705 0.819566 . . #POSITIVE

SEQUENCE netOGlyc-4.0.0.13 CARBOHYD 708 708 0.933984 . . #POSITIVE

SEQUENCE netOGlyc-4.0.0.13 CARBOHYD 716 716 0.937569 . . #POSITIVE

SEQUENCE netOGlyc-4.0.0.13 CARBOHYD 722 722 0.966047 . . #POSITIVE

SEQUENCE netOGlyc-4.0.0.13 CARBOHYD 725 725 0.820749 . . #POSITIVE

SEQUENCE netOGlyc-4.0.0.13 CARBOHYD 726 726 0.938729 . . #POSITIVE

SEQUENCE netOGlyc-4.0.0.13 CARBOHYD 732 732 0.911842 . . #POSITIVE

SEQUENCE netOGlyc-4.0.0.13 CARBOHYD 739 739 0.685243 . . #POSITIVE

SEQUENCE netOGlyc-4.0.0.13 CARBOHYD 745 745 0.908688 . . #POSITIVE

SEQUENCE netOGlyc-4.0.0.13 CARBOHYD 750 750 0.474997 . .

SEQUENCE netOGlyc-4.0.0.13 CARBOHYD 759 759 0.472408 . .

SEQUENCE netOGlyc-4.0.0.13 CARBOHYD 766 766 0.572043 . . #POSITIVE

SEQUENCE netOGlyc-4.0.0.13 CARBOHYD 771 771 0.484373 . .

SEQUENCE netOGlyc-4.0.0.13 CARBOHYD 782 782 0.631361 . . #POSITIVE

SEQUENCE netOGlyc-4.0.0.13 CARBOHYD 787 787 0.854085 . . #POSITIVE

SEQUENCE netOGlyc-4.0.0.13 CARBOHYD 791 791 0.638541 . . #POSITIVE

SEQUENCE netOGlyc-4.0.0.13 CARBOHYD 840 840 0.871923 . . #POSITIVE

SEQUENCE netOGlyc-4.0.0.13 CARBOHYD 843 843 0.866972 . . #POSITIVE

SEQUENCE netOGlyc-4.0.0.13 CARBOHYD 850 850 0.791898 . . #POSITIVE

SEQUENCE netOGlyc-4.0.0.13 CARBOHYD 855 855 0.938474 . . #POSITIVE

SEQUENCE netOGlyc-4.0.0.13 CARBOHYD 886 886 0.744451 . . #POSITIVE

SEQUENCE netOGlyc-4.0.0.13 CARBOHYD 889 889 0.775707 . . #POSITIVE

SEQUENCE netOGlyc-4.0.0.13 CARBOHYD 890 890 0.948924 . . #POSITIVE

SEQUENCE netOGlyc-4.0.0.13 CARBOHYD 892 892 0.823935 . . #POSITIVE

SEQUENCE netOGlyc-4.0.0.13 CARBOHYD 893 893 0.911617 . . #POSITIVE

SEQUENCE netOGlyc-4.0.0.13 CARBOHYD 897 897 0.689014 . . #POSITIVE

SEQUENCE netOGlyc-4.0.0.13 CARBOHYD 910 910 0.916762 . . #POSITIVE

SEQUENCE netOGlyc-4.0.0.13 CARBOHYD 914 914 0.790114 . . #POSITIVE

**FAT Domain**

SEQUENCE netOGlyc-4.0.0.13 CARBOHYD 920 920 0.557181 . . #POSITIVE

SEQUENCE netOGlyc-4.0.0.13 CARBOHYD 929 929 0.305129 . .

SEQUENCE netOGlyc-4.0.0.13 CARBOHYD 939 939 0.432465 . .

SEQUENCE netOGlyc-4.0.0.13 CARBOHYD 940 940 0.522373 . . #POSITIVE

SEQUENCE netOGlyc-4.0.0.13 CARBOHYD 963 963 0.147894 . .

SEQUENCE netOGlyc-4.0.0.13 CARBOHYD 967 967 0.278502 . .

SEQUENCE netOGlyc-4.0.0.13 CARBOHYD 971 971 0.449772 . .

SEQUENCE netOGlyc-4.0.0.13 CARBOHYD 978 978 0.254878 . .

SEQUENCE netOGlyc-4.0.0.13 CARBOHYD 979 979 0.431879 . .

SEQUENCE netOGlyc-4.0.0.13 CARBOHYD 992 992 0.0186447 . .

SEQUENCE netOGlyc-4.0.0.13 CARBOHYD 999 999 0.0566395 . .

SEQUENCE netOGlyc-4.0.0.13 CARBOHYD 1010 1010 0.0612554 . .

SEQUENCE netOGlyc-4.0.0.13 CARBOHYD 1011 1011 0.128537 . .

SEQUENCE netOGlyc-4.0.0.13 CARBOHYD 1022 1022 0.0718828 . .

SEQUENCE netOGlyc-4.0.0.13 CARBOHYD 1049 1049 0.119378 . .
