# Supplementary figures and images for: O-GlcNAcylation of Focal Adhesion Kinase Regulates Cell Adhesion, Migration, and Proliferation via the FAK/AKT Pathway
Source: Biomolecules. 2024 Dec 10;14(12):1577. doi: 10.3390/biom14121577 (PMC11674061; doi:10.3390/biom14121577)

# Original WB Figures

**Fig 1B**

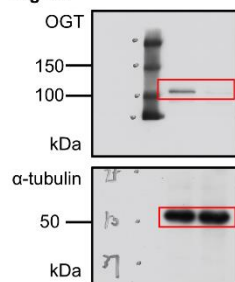

**Fig 1C**

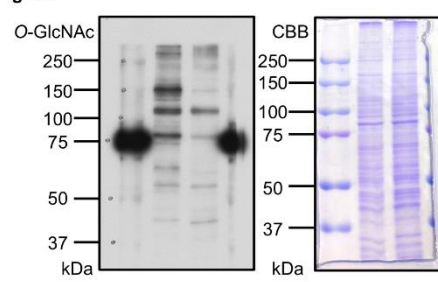

**Fig 1D**

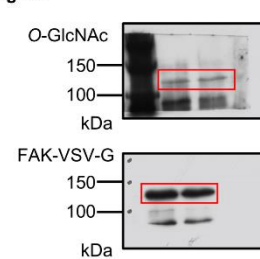

**Fig 2A**

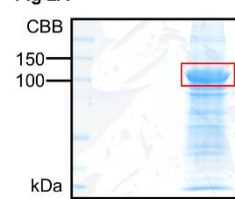

**Fig 3A**

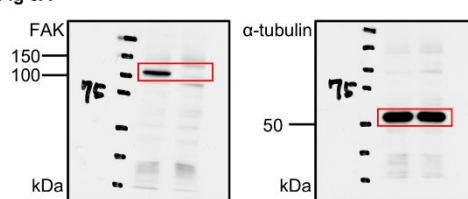

**Fig 3C**

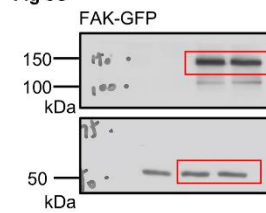

**Fig 3B**

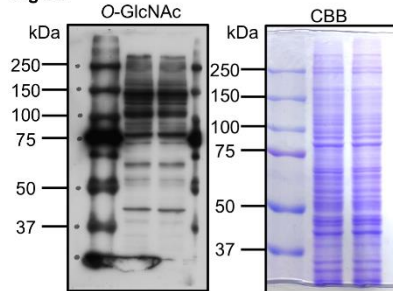

**Fig 3E**

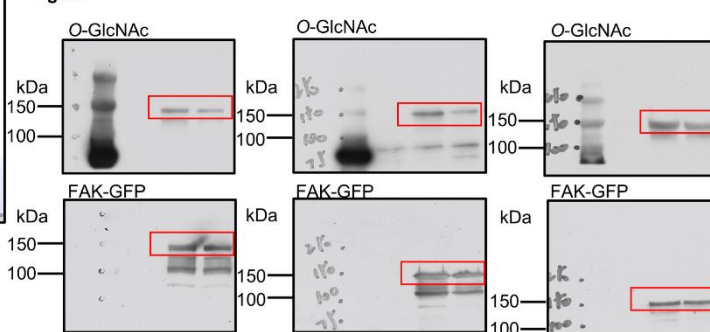

**Fig 3D**

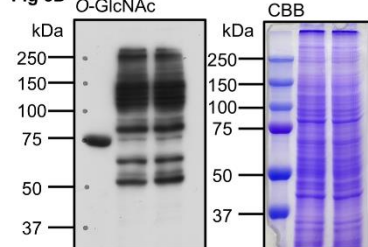

**Fig 5A**

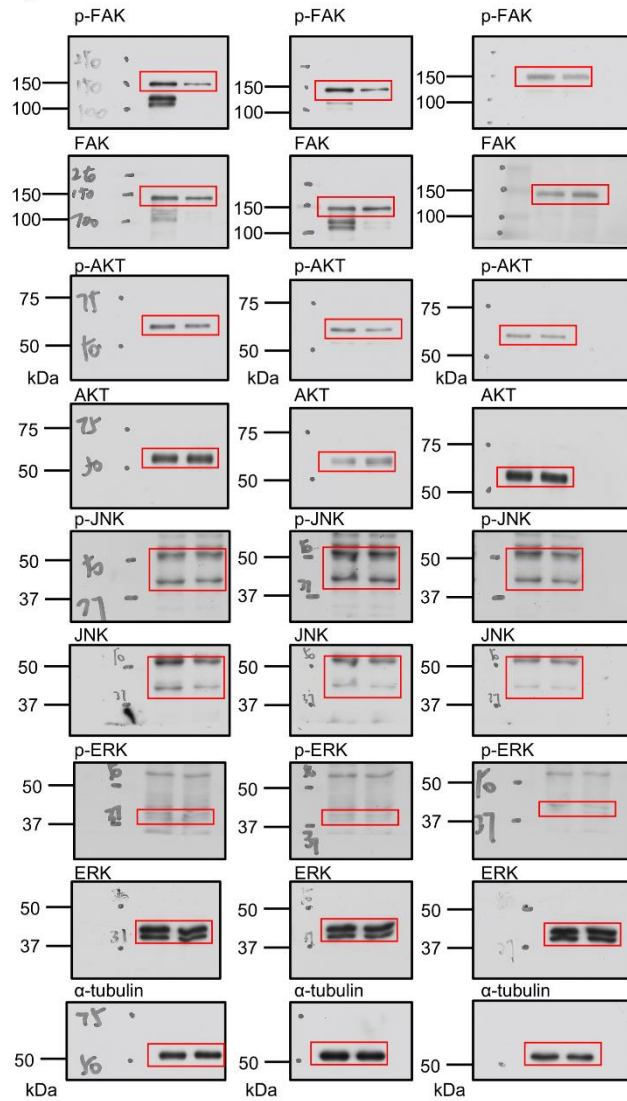

**Fig 5B**

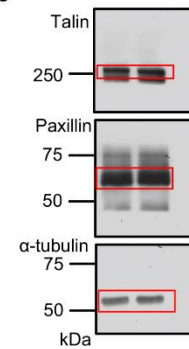

**Fig 5C**

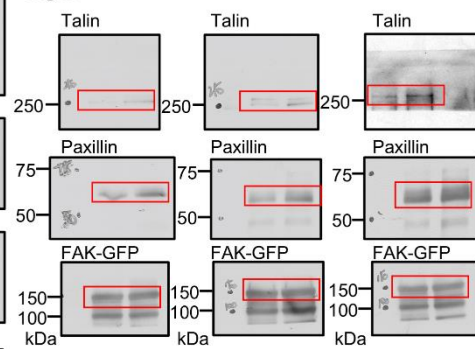

Supplement: Supplementary file 1 [file biomolecules-14-01577-s001.zip › biomolecules-3317003- original WB figure.pdf]
